# Case report: Minimally invasive excision of multifocal cardiac papillary fibroelastomas involving right atrium and aortic valve

**DOI:** 10.3389/fcvm.2022.908567

**Published:** 2022-08-03

**Authors:** Peng Teng, Peng Hu, Shuai Yuan, Liang Ma

**Affiliations:** ^1^Department of Cardiovascular Surgery, The First Affiliated Hospital, College of Medicine, Zhejiang University, Hangzhou, China; ^2^Department of Echocardiography and Vascular Ultrasound Center, The First Affiliated Hospital, College of Medicine, Zhejiang University, Hangzhou, China

**Keywords:** cardiac papillary fibroelastoma, minimally invasive, multifocal, right atrium, aortic valve, cardiac tumor

## Abstract

**Background:**

Cardiac papillary fibroelastomas (CPFs) are rare benign cardiac tumors most commonly found on left-sided cardiac valves. Right atrial CPFs are extremely rare, accounting for only 2% of all CPFs. Median sternotomy is a typical approach for surgical excision of CPFs in most cases. Herein, we report an extremely rare case of multifocal CPFs involving the right atrium and aortic valve that were surgically excised *via* minimally invasive right anterolateral thoracotomy.

**Case Summary:**

A 59-year-old Chinese man was admitted because of an incidental finding of a right atrial mass on transthoracic echocardiography during a routine check-up. The mass was initially diagnosed as a myxoma, and the patient was scheduled for minimally invasive excision *via* right anterolateral thoracotomy. An additional mass on the non-coronary cusp of the aortic valve was identified using intraoperative transesophageal echocardiography. The patient still underwent complete tumor excision *via* right anterolateral thoracotomy. Both neoplasms were pathologically diagnosed as CPFs.

**Conclusions:**

This case highlights the need for a comprehensive cardiac evaluation of cardiac tumors because CPFs can manifest as multifocal lesions. Moreover, minimally invasive surgery is highly feasible as the CPF can be easily excised, and the valve can usually be preserved.

## Introduction

Cardiac papillary fibroelastomas (CPFs) are rare and considered one of the most common benign cardiac tumors (1). The incidence of CPFs is approximately 1,380/100 million individuals and constitutes 11.5% of all primary cardiac tumor (2). Although CPFs may arise on any endocardium-lined surface, more than three-quarters of CPFs occur on valvular surfaces, with left-sided valves being more commonly affected than those on the right. Right atrial CPFs are extremely rare and account for only 2% of all CPFs (3). Multifocal CPFs with simultaneous involvement of the right atrium (RA) and the aortic valve (AV) have seldom been reported. Moreover, most cases of multifocal CPFs were treated with surgical excision *via* a median sternotomy. Herein, we present a case of minimally invasive excision of multifocal CPFs involving the RA and the AV.

## Case presentation

### History of presentation and investigation

**Figure 1:**
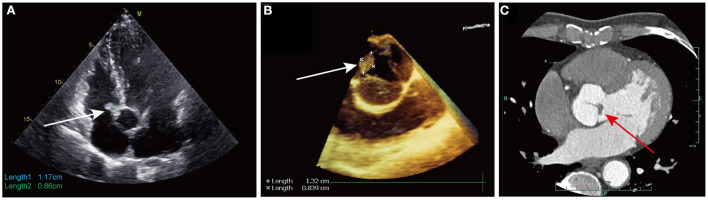
**(A)** Transthoracic echocardiography showed an irregular hyperechoic mobile mass (1.2 × 0.9 cm in size, white arrow) with a stalk arising from the right atrium adjacent to the aortic root; **(B)** Transesophageal echocardiography showed an additional mobile mass (1.3 × 0.8 cm in size, white arrow) on the ventricular side of the non-coronary cusp of aortic valve; **(C)** Coronary computed tomography angiography showed abnormal thickness of non-coronary cusp of aortic valve, which represented the cardiac papillary fibroelastoma (red arrow) on the aortic valve.

A 59-year-old Chinese male was admitted because of incidental findings of a right atrial mass on transthoracic echocardiography (TTE) during a routine check-up. No medical history has been reported to date. The laboratory results and physical examination findings were unremarkable. TTE revealed an irregular hyperechoic mobile mass (1.2 × 0.9 cm in size) with a stalk arising from the RA adjacent to the aortic root (Figure 1A). TTE also revealed thickened leaflets of AV, which was considered to be a degenerative change. No wall motion or other structural valve abnormalities were reported. Coronary computed tomography angiography (CTA) demonstrated no obvious stenosis of the coronary artery.

### Diagnosis

The right atrial mass was initially diagnosed as myxoma or papillary fibroelastoma.

### Management

**Figure 2:**
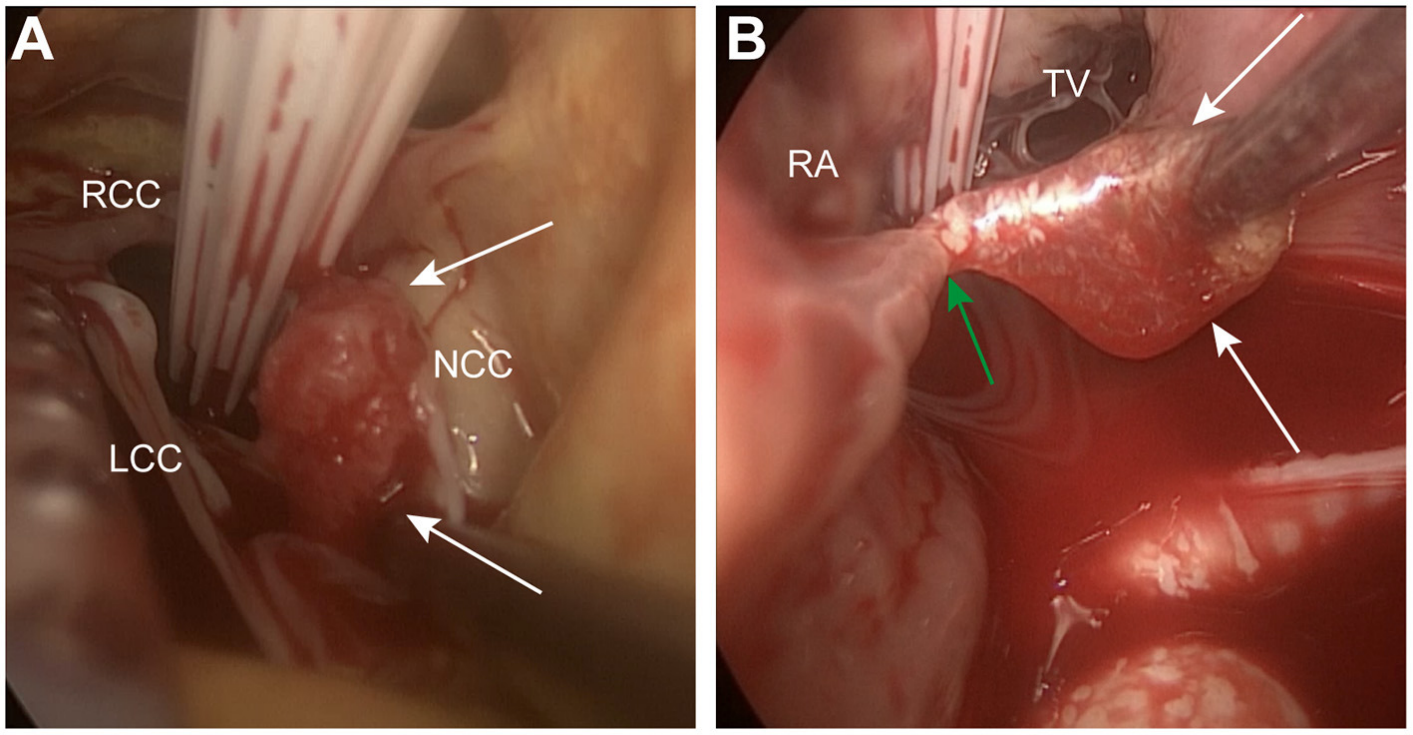
**(A)** CPF was located on the ventricular side of non-coronary cusp of aortic valve (white arrows); **(B)** CPF (white arrows) was located in the RA adjacent to aortic root with a stalk (green arrow). CPF, cardiac papillary fibroelastoma; NCC, non-coronary cusp; RCC, right-coronary cusp; LCC, left-coronary cusp; RA, right atrium; TV, tricuspid valve.

The patient was scheduled for minimally invasive excision of the right atrial mass *via* right anterolateral thoracotomy. Intraoperative transesophageal echocardiography (TEE) after anesthesia detected an additional mobile mass (1.3 × 0.8 cm in size) on the ventricular side of the non-coronary cusp of the AV (Figure 1B). Both right atrial and aortic valvular masses were considered CPFs due to their stippled edge and good mobility with stalks. Considering the high probability of AV preservation in cases of primary surgical indication for CPF based on our experience, we proceeded with the surgery *via* right anterolateral thoracotomy, without conversion to media sternotomy. Cardiopulmonary bypass was established through the femoral artery and vein cannulation. A 5 cm right mini-thoracotomy incision was made in the fourth interspace, which started medially to the anterior axillary line. Cardioplegia was achieved by antegrade perfusion of cold blood cardioplegia solution through a Y-shaped cannula. After transverse aortotomy, the tumor was located on the ventricular side of the noncoronary cusp of the AV (Figure 2A) and was easily excised by shaving its stalk. AV leaflet inspection confirmed their anatomical preservation. The aorta was then closed, and the heart resumed beating automatically. Then the tip of the cannula in the inferior vena cava was laid in the right atrium, 1 cm above the inferior atriocaval junction. No caval veins were snared and after transverse right atriotomy, a vacuum-assisted venous drainage controller was used to maintained the negative pressure ranging from−20 to−30 mmHg, or according to the blood level in the right atrium. The right atrial tumor (Figure 2B) was successfully excised on a beating heart by shaving its stalk (Supplementary Video 1). TEE revealed trivial AV regurgitation and complete excision of the masses. As only still echo images were regular archived, we retrospectively found abnormal thickness of non-coronary cusp of AV on coronary CTA, representing the CPF which was misdiagnosed as degenerative thickening of leaflet (Figure 1C). The postoperative course was uneventful and the patient was discharged on postoperative day six. After a 2-year follow-up, TTE showed no signs of CPF recurrence.

### Macroscopic observation and histopathological examination

**Figure 3:**
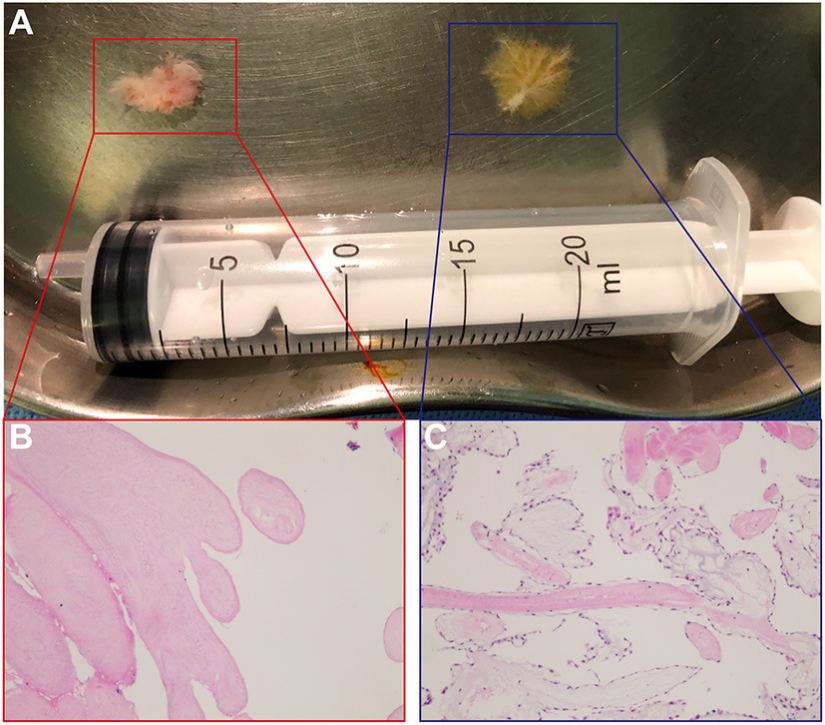
**(A)** Macroscopic inspection of the tumors when immersed in water showed a “sea anemone” phenomenon. On the left is the tumor on the aortic valve, and on the right is the tumor in the right atrium. **(B,C)** Histopathologic examination of the tumors [panel **(B)** represents the tumor on the aortic valve and panel **(C)** represents the tumor in the right atrium] with hematoxylin-eosin staining (100×) confirmed the diagnosis of cardiac papillary fibroelastomas.

When immersed in water, both tumors showed “the sea anemone” phenomenon, which strongly suggested the presence of CPFs (Figure 3A). Both tumors were fixed with formalin, embedded with paraffin and stained with hematoxylin and eosin. Histopathological examination demonstrated bundles of papillary projections with a collagenous core surrounded by a single layer of endothelium, and a final diagnosis of multifocal CPFs was made (Figures 3B,C).

## Discussion

Our study reported an extremely rare case of multifocal CPFs involving the RA and AV that was successfully treated with complete tumor excision *via* right anterolateral thoracotomy, of which the surgical approach has seldom been reported. This case highlights two important points: (1) CPFs could present as multifocal involvement and should be comprehensively evaluated by multimodality imaging, and (2) a minimally invasive approach is suitable for surgical excision of multifocal CPFs based on the fact that the valve can usually be preserved when tumor excision is the primary surgical indication (4).

Despite CPF being one of the top three common primary benign cardiac tumors, the other two being myxomas and lipomas (2, 5) quite a few scholars consider it to be the most common (6). Moreover, it is the most common primary cardiac valvular tumor, whereas non-valvular involvement is infrequent. Right-sided CPFs only account for ~10% of cases, and the tricuspid valve is the most common right-sided location (7). Right atrial CPFs are rare, accounting for only 2% of all CPFs, which makes our case of multifocal CPFs involving the RA and AV extremely rare.

CPFs are commonly found incidentally in asymptomatic individuals (8). When symptoms develop, the most common are systemic (9) or pulmonary embolization (10), followed by coronary ostium obstruction (5), heart failure, and even sudden cardiac death due to the detachment of tumor fragments or thrombi related to the tumor. Right-sided CPFs tend to be larger at presentation than left-sided CPFs (4), possibly due to their tendency to present late as they have fewer clinical implications. Typical CPF exhibit a “frond-like” appearance because of its papillary projections arising from the fibrous stalk attached to the endocardium, which makes it look like a sea anemone when immersed in water. On TEE, the borders may appear slightly stippled or shimmering caused by vibration at the tumor-blood interface because of its finger-like projections. TEE is more sensitive than TTE because of the typical small size of CPFs, and about one-quarter of patients with CPFs might be detected on TEE but not TTE (4), similar to our case where the CPF on the AV was misdiagnosed by preoperative TTE.

Histologically, CPFs are composed of thickened, broad papillae of varying lengths, lined by endothelial cells, with avascular connective tissue stroma, myxoid, fibrosis or collagen. Immunohistochemically, the cells covering the surface are positive for factor VIII-related antigen and CD 34, in keeping with their presumed vascular endothelial origin, but also the S-100 protein may be positive (11).

Surgical indications for CPFs remain controversial. The mainstream view recommends surgical excision in patients who are good surgical candidates with a large (≥1 cm) or mobile left-sided CPF (12). Asymptomatic right-sided CPFs can be managed more conservatively, unless they are associated with embolization. Surgical excision *via* median sternotomy is considered the standard surgical approach, particularly in cases of multifocal CPFs (13). However, based on the experience of our center and other studies (4), valves are usually spared when tumor excision of the CPFs is the primary surgical indication. Therefore, we highlight the importance and feasibility of minimally invasive excision *via* right anterolateral thoracotomy, as most patients with CPFs are asymptomatic and surgical damage should be minimized. In our case, both CPFs were easily excised by simple shave excision, and valvular function was unaffected. However, long-term antiplatelet treatment is recommended for patients who are not candidates for surgery or who refuse surgery.

## Conclusions

CPFs can present as multifocal lesions and should be comprehensively evaluated using multimodal imaging. TEE is more sensitive than TTE for cardiac mass detection. As valves are usually spared when CPF excision is the primary surgical indication, a minimally invasive approach *via* right anterolateral thoracotomy is recommended to minimize surgical damage, especially in asymptomatic patients.

## Data availability statement

The original contributions presented in the study are included in the article/Supplementary material, further inquiries can be directed to the corresponding author.

## Ethics statement

Written informed consent was obtained from the participant/s for the publication of this case report. Written informed consent was obtained from the individual(s) for the publication of any potentially identifiable images or data included in this article.

## Author contributions

LM: conceptualization. PT and PH: data collection and analysis. PT: writing. SY: writing, review, and editing. All authors have approved the manuscript for publication.

## Funding

This study was funded by the Key Research and Development Program of Zhejiang Province, China (Project Number: 2019C03008) and the Natural Science Foundation of Zhejiang Province, China (Project Number: LQ22H020005).

## Conflict of interest

The authors declare that the research was conducted in the absence of any commercial or financial relationships that could be construed as a potential conflict of interest.

## Publisher's note

All claims expressed in this article are solely those of the authors and do not necessarily represent those of their affiliated organizations, or those of the publisher, the editors and the reviewers. Any product that may be evaluated in this article, or claim that may be made by its manufacturer, is not guaranteed or endorsed by the publisher.

## Supplementary material

Supplementary Video 1 The video of minimally invasive tumor excision *via* right anterolateral thoracotomy recorded by endoscope.

## Abbreviations

CPF: cardiac papillary fibroelastoma
TTE: transthoracic echocardiography
CTA: computed tomographic angiography
TEE: transesophageal echocardiography
RA: right atrium
AV: aortic valve.
